# Role of diet and dietary habits in causing dental caries among adults reporting to a tertiary care hospital in Pakistan; a case-control study

**DOI:** 10.1016/j.heliyon.2023.e23117

**Published:** 2023-11-30

**Authors:** Kiran Javed, Muhammad Zubair Nasir, Maham Jalees, Manzoor Ahmed Manzoor

**Affiliations:** a Rawal Institute of Health Sciences, Islamabad, Pakistan; b Shifa College of Dentistry, Islamabad, Pakistan

**Keywords:** Adults, Dental caries, Diet, Dietary habits, Refined sugar, Frequency of sugar consumption, Smoking

## Abstract

**Objective:**

To determine the association of diet and dietary practices with dental caries among adults.

**Design:**

A case-control study.

**Setting:**

Operative Department, Rawal Institute of Health Sciences, Islamabad, Pakistan.

**Participants:**

300 participants of both genders, aged 25–50 years.

**Interventions:**

A food frequency questionnaire and a patient proforma were used to determine the frequency and preferences of diet and dietary habits that may be associated with dental caries among adults, respectively. The diet and dietary habits of 150 adults with caries (cases) were compared with those of 150 adults without dental caries (control). An independent sample T-test was applied to determine the difference in mean age. Mann-Whitney and Chi-Square tests were applied to determine the significance of diet and dietary habits respectively. Multivariate logistic regression analysis determined the odd ratio change in significant variables. *P*-value ≤0.05 was considered significant.

**Results:**

Refined sugar (p-value = 0.69), fruit juices (p-value = 0.45), carbonated beverages (p-value = 0.91), duration of consumption of sugary food (p-value = 0.07), and frequency of brushing (p-value = 0.15) were not found to be significantly associated with dental caries in adults. The gender (p-value = 0.02), preferred time for eating sugary foods (p-value <0.001), smoking (p-value <0.001), and tea consumption (p-value = 0.02) were found to be significantly associated with dental caries.

**Conclusion:**

Adults who regularly consumed sugar as a snack other than regular mealtimes were more likely to be associated with dental caries. Men, smokers, and adults who frequently took shots of sugar with their tea were more likely to be associated with dental caries.

## Strength of the study

•Limited research has been conducted globally on adults to determine how diet and dietary habits are linked to dental caries. Therefore, the current study addresses this issue among adults.
•Dental caries is a complex, multifactorial disease. So, to conduct an effective study for determining the association of diet and habits with dental caries, the inclusion and exclusion criteria were developed to minimize the confounding factors.
•This study provides a holistic view of the association of diet and dietary habits with the emergence of dental caries in one glance.

## 1 Introduction

Dental caries is a non-communicable disease with a global prevalence of 35 % for all ages combined. It is a biofilm-mediated, sugar-driven, multifactorial, dynamic disease caused by increased demineralization of dental hard tissues [1].

Diet, oral health, and general health are interlinked with each other. A diet high in refined sugar and carbonated drinks can cause dental caries and erosion, which can affect dietary intake due to discomfort while eating. This selective eating results in a deficiency of many nutrients in the body, which are essential for the integrity of the whole body and teeth, surrounding tissues, bones, and wound healing [2]. Thus, a good diet is a fundamental component of good oral health and overall health.

Studies on the association of diet with dental caries have primarily been conducted on children, although adults are more affected than children. Worldwide, the World Health Organization (WHO) estimated that 2 billion people have permanent tooth decay, and 514 million children have primary tooth decay [3]. Limited studies have reported this relationship among adults worldwide. A thorough literature search revealed that, as of this point, no such study has been carried out on Pakistani adults.

Bernabé E. and Sheiham A. in 2014 reported that adults were 500 % more likely to experience dental caries than children. This age-period-and-cohort (APC) research suggested that significant levels of caries in adults were seen despite low levels of caries in children in four developed countries, including England and Wales, Sweden, the USA, and Japan [4]. Another longitudinal study also found that tooth caries levels in adults were greater even though preventive measures were provided in childhood and adolescence [5]. The results motivated the authors to assess dental caries in adults.

Patients who visited the current setting of the study for treatment often inquired about the relationship between diet and dental caries, which served as the impetus for this study. One of their frequently asked questions was why they had dental caries despite regular brushing of teeth and limited sugar consumption aside from what was put in their tea.

The likelihood of developing dental caries is greatly influenced by a variety of medical disorders, genetic variations, and extended pharmacological treatments that might cause hyposalivation [[6], [7], [8]]. The key risk factors, according to the WHO, are inadequate oral hygiene, lack of health education, fluoride intake, and sociodemographic factors in addition to increased consumption of cariogenic foods, frequency and type of diet, and snacking behavior. Cigarettes, alcohol, and an unhealthy diet are additional risk factors for oral illnesses [9,10].

This study aimed to assess the relationship of diet and dietary practices with dental caries. The null hypothesis of the current study was ‘‘there is no association between dental caries and diets such as refined sugar, fruit juices, and carbonated drinks among adults’’. Moreover, ‘‘there is no association between dental caries and dietary habits like smoking, brushing, snacking, and the duration of consumption of sugary foods among adults’’.

## 2 Materials and methods

**Study design and setting:** A retrospective, hospital-based case-control study was conducted on adults who worked at Rawal Institute of Health Sciences (RIHS), as well as those attending the Operative department from January 1, 2020 to July 15, 2021 after approval from the ethical committee of RIHS (reference #RIHS/IRB/19–005). Adults with dental caries were assigned to the case group, whereas the control group comprised adults without dental caries. Group matching in the ratio of 1:1 was performed between the participants in the case and control groups in terms of age (5 years caliper), education, socioeconomic level, water fluoridation, oral hygiene, health, and medication use. By selecting the patients who came to the hospital for checkups as cases and their relatives or attendants as controls, the selection process was made simpler. In the same way, when hospital staff members were involved, the controls were also identified among them.

### 2.1 Inclusion criteria

Minimizing the confounding variables by group matching, the inclusion criteria involved middle-class adults between the ages of 25 and 50, both genders, who had completed at least secondary school, lived in the RIHS neighborhood, had acceptable oral hygiene (i.e., brushed their teeth at least once daily), and did not have a significant calculus deposit (0 and 1 calculus index scale).

### 2.2 Exclusion criteria

Adults who did not regularly brush their teeth, had large calculus deposits, dry mouth or fissured tongue, caries due to the radiation or any syndrome, parafunctional habits like bruxism, or history of frequently swishing water in their mouth, were immune compromised or mentally compromised, and had used steroids or other medications for a longer period, were excluded from the study. This further minimalizes the remaining confounding variables.

### 2.3 Study instruments

Adults who were willing to participate in the study were briefed about the study and informed verbal and written consent (Appendix 1) were obtained from each participant. Information about diseases and medication was obtained from medical history. Clinical/oral examination indicated the caries, calculus index scale (0 and 1), and fissured tongue (indication of hyposalivation) with the help of supplementary tools like mirror, probe, tweezer, good light, and radiographs. Participants’ proforma (Appendix 2) included demographic details like age, gender, education, address, and determinants of socioeconomic status. It also included smoking, consumption of alcohol, beetle, and snuff, as well as dietary habits like duration of food consumption, consuming sugary food with or without meals, and brushing frequency. The food frequency questionnaire (FFQ, Appendix 3) was used as a retrospective data collection tool, having nine mutually exclusive categories ranging in order from “never or less than a month” to “six or more times per day” for consumption of different dietary items like refined sugar, carbonated or fizzy drinks, fruit juices, and tea. Patients may miss out when asking about refined sugar consumption more broadly. So, for easy recall of refined sugary food, the FFQ was developed with the names of commonly used items containing sugar in Pakistan, like ice cream, chocolate, mithai/halwa, candies/jellies, jam, biscuits, cake/muffins/pastries/doughnuts, breath mints, and sugary chewing gums.

The source of FFQ of the current investigation was from a similar study conducted on the adult Pakistani urban population of both genders. The study that validated this FFQ among Pakistanis suggests that it can be used globally and, in all languages, [11].

Adults having one or more visibly cavitated carious teeth, i.e., ICDAS 3, 5, and 6 (International Caries Detection and Assessment System), as well as those having black shadows under non-cavitated enamel and involving dentin (ICDAS-4, as observed from radiographs), were considered carious. Adults who had nil to mild calculus, i.e., having 0 and 1 calculus index scale, were acceptable for the study.

### 2.4 Sample size calculation

Since limited work has been done regarding finding the prevalence of dental caries in Pakistan, a meta-analysis to determine the prevalence of dental caries in neighbor country India was used to determine the sample size. The study showed that the prevalence of dental caries in Indian adults is increasing from 60 % to 78 % [12]. The calculation of sample size was done by applying the following formula.

n = z^2^p (1-p)/e^2^, where

p=78%=78/100=0.78,1−p=22%=22/100=0.22,z=95%=1.96,e=5%=5/100=0.05.

$$n=\left(1.96^{2}\times 0.78\times 0.22\right)/\;0.05^{2}=263$$

The sample size calculated was 263; however, it was rounded off to 300. So, 150 adults with caries were selected as cases whose diet and other dietary habits were compared with the control group of 150 adults without caries. The data was collected personally after a briefing on the objectives of the study. The same questionnaire was filled out by adults from both groups to get a comparison of whether the dental caries was due to differences in diet or dietary habits. The focus was to add all the common sugary foods for easy recall.

### 2.5 Statistical analysis

The mutually exclusive categorical data obtained from the FFQ, and data obtained from participants' proforma of 300 adults were then entered in SPSS version 20 for data analysis. An ‘independent sample T-test’ was applied to determine the difference in mean age between the two groups. For determining the relationship between dietary habits and dental caries, the ‘Chi-Square test’ was applied. The total frequency of refined sugar was obtained by adding the frequencies of all the items containing refined sugar. Since it was continuous data, the normality was checked by using the one-sample Kolmogorov-Smirnov Test’. The results showed that the data was not distributed normally (p-value <0.001) so, the non-parametric ‘Mann-Whitney *U* Test’ was applied to compare the diets of adults with dental caries to caries-free adults. Multivariate regression analysis was also applied by using caries as a dependent variable, and items of diet and dietary habits as the independent variables. *P*-value <0.05 was considered statistically significant.

Kaiser–Meyer–Olkin (KMO) test was used to determine sample adequacy. A KMO value of >0.7 was considered sufficient for the sample size chosen. The response of the participants was used to run a Cronbach's alpha test to evaluate the FFQ's internal consistency. The value of Cronbach's alpha test above 0.6 was considered acceptable to determine the reliability of FFQ.

### 2.6 Patient and public involvement

There was no patient and public involvement in designing, conducting, or reporting the plan of this study.

## 3 Results

According to Table 1, the mean age of adults in the case and control groups was 33.0 + 6.3 and 35.7 + 6.3, respectively. The independent sample T-test resulted in a p-value of 0.717, indicating that the two groups' ages were effectively matched since there was no statistically significant difference in their mean ages.

**Table 1:** Descriptive analysis of mean age of carious and non-carious adults.

Groups	Minimum age	Maximum age	Mean age	±SD	p-value
Carious	25	50	33.0	6.3	0.717
Non-carious	25	50	35.7	6.3	

Gender analysis in Table 2 suggests that within the cases, there were 87 males (58 %) and 63 females (42 %), as opposed to 66 males (44 %) and 84 females (56 %) in the control group. The distribution of males and females was significantly different among cases and the control group (p-value = 0.02). The number of males was found to be significantly higher among the case group as compared to that among control groups.

**Table 2:** Inferential Analysis of duration of consumption of sugary food, time of frequent consumption of sugary food, smoking, and gender.

Inferential Analysis (Chi-Square Test)					p-value
Gender	87 (5 %) Carious Male	63 (42 %) Carious Female	66 (56 %) Non-Carious Male	84 (44 %) Non-Carious Female	0.02
Smoking	76 % Carious Smokers	24 % Carious Non-smokers	6 % Non-Carious Smokers	94 % Non-Carious Non-Smokers	<0.001
Frequency of consumption of sugar food.	47 % Carious with meal	53 % Carious Between meals	62 % Non-Carious with meal	38 % Non-Carious Between meals	<0.001
Duration of consumption of sugary food.	21.9 % Carious Slow Consumption	78.1 % Carious Quick Consumption	27.7 % Non-Carious Slow Consumption	72.3 % Non-Carious Quick Consumption	0.07
Brushing frequency per day	Once	49 [32.6 %] Carious	44 [29.3 %] non carious		0.176
	Twice	87 [58 %] Carious	97 [64.7 %] non carious		
	Thrice	14 [9.3 %] Carious	9 [6 %] non carious		

According to Table 2, the relationship between smoking and dental caries was also determined to be significant, meaning that smokers were more common among the case group as compared to the control group (p-value <0.001). Only one individual from the case group accepted alcohol consumption. None of the participants mentioned the use of snuff, betel, or betel nuts.

Table 2 displays a strong correlation (p-value <0.001) between dental caries and the time of frequent sugary food consumption. The case group (53 %) had a higher liking for taking sweets between regular meals compared to the control group (47 %).

Furthermore, Table 2 revealed that the preferred brushing timings (p-value = 0.154), frequency of brushings per day (p-value = 0.176), and duration of consumption of sugary or carbonated drinks (p-value = 0.07) were not statistically significant. This suggests that adults in both groups practiced these habits almost equally.

Table 3 revealed that the relationship between dental caries and refined sugar (p-value = 0.69), fruit juice (p-value = 0.91), and carbonated beverages (p-value = 0.45) was not significant. It can be inferred that participants in both groups equally consumed these dietary items.

**Table 3:** Mann- Whitney test for the comparison of frequency of food consumption by carious and non-carious adults.

Food	Carious [n = 150]	Non-Carious [n = 150]	p-value
	Mean Rank	Mean Rank	
Refined sugar	148.52	152.48	0.69
Fruit juice	149.93	151.07	0.91
Carbonated drink	153.89	147.11	0.45
Tea	161.42	139.58	0.02

The difference in mean rank for tea consumption was found to be statistically significant (p-value = 0.02, Table 3), which suggests that the cases consumed more tea than the controls. Multivariate logistic regression analysis suggests no significant correlation between dental caries and refined sugar, fruit juices, fizzy drinks, tea, brushing frequency, and duration of consumption of sugary food (p-value ≥0.05). Smoking and snack time were found to be significant (p-value <0.05). As shown in Table 4, the odd ratio and confidence interval at 2.5 % and 97.5 % of smoking (OR = 6.048, CI = 2.536, 14.426) and snack time (OR = 14.167, CI = 4.142, 48.456) were found to be significant (p-value <0.05).

**Table 4:** Multivariate regression analysis showing odd ratio and Confidence Interval at 2.5 % and 97.5%of Significant Variables.


	B	S.E.	Wald	df	p-value	Exp(B)	95 % C.I. for EXP(B)	
							Lower	Upper
Smoking	1.800	.443	16.469	1	<0.001	6.048	2.536	14.426
snack time	2.497	.641	15.182	1	<0.001	12.145	3.459	42.647
Constant	−3.041	.610	24.871	1	<0.001	.048		
a. Variable(s) entered on step 1: snack time								
b. Variable(s) entered on step 2: smoking								

The multivariate logistic regression displayed 94 % sensitivity and 74 % specificity. The precise overall percentage was 84 %. The KMO sampling adequacy score of 0.774 indicates that a sample size of 300 adults is suitable. The FFQ's internal consistency and reliability as demonstrated by Cronbach's alpha is 0.814 which suggests that the FFQ has very good reliability.

## 4 Discussion

Dental caries is multifactorial [1]. The goal of the current study was to reduce the confounding factors as much as possible to identify the true role of diet and dietary habits associated with dental caries. The top age limit was decided to be 50 years to prevent recall bias [13], and the lower age limit was restricted since habits become more stable after the age of 25 years. The participants of the study were locals of the RIHS neighborhood, suggesting that they lived in an area with the same water fluoride levels. This helps in reducing the bias that different levels of fluoride concentration are associated with different caries severity [14]. Calculus is the niche for bacteria, which develops because of poor oral hygiene [15]. The calculus index scale was kept to a minimum because the primary cause of tooth decay is acidogenic bacteria. Thus, poor oral hygiene was not considered in the study to determine the genuine role of diet and dietary habits, including effective brushing versus frequency of brushing, in contributing to dental caries. Participants' occupations, estimated incomes, educational institutions attended by themselves or their children, the number of dependents in the household, ownership of a car, and whether they lived in a rented or owned home were all factors used to define the socioeconomic class [16].

The current study suggests that males are at higher risk of dental caries as compared to females. The reason behind this may be that males smoke more as compared to females [17].

Cigarettes contain nicotine, which promotes the growth of cariogenic bacteria like Streptococcus mutans, Lactobacilli, Candida albicans, and Actinomyces. Smoking alters the chemical and bacterial components as well as lowers the buffering ability of saliva, and therefore promotes a caries-susceptible environment [18]. Moreover, nicotine encourages the attachment of Streptococcus mutans to the surface of the tooth and further boosts the occurrence and severity of dental caries. Thus, the risk factor for smoking-induced caries might be nicotine [19]. The strong association between smoking and dental caries in the current study means smokers are more at risk for dental caries as compared to non-smokers. The binary logistic regression analysis suggests that with every unit increase in smoking, the chance of increasing dental caries is 6.048. The same perspective is supported by Khan et al. who concluded that smokers aged 35 years revealed considerably more DMFT than non‐smokers [20].

The consumption of sugary foods during meals suggests that adults prefer to eat sweetened food at mealtime along with other food. However, the consumption of sugary food between meals indicates that adults prefer to take sugar-rich food as a snack at a time other than regular mealtimes. The current study suggests that adults who take more sugary food between meals are at greater risk of dental caries than adults without dental caries. The binary logistic regression analysis also suggests that with every unit increase in snaking, the chance of increasing dental caries will be 12.145. These findings are supported by Chankanka, who concluded that fruit juices and beverages consumed between meals as snacks are associated with dental caries [21]. A study conducted on children with caries in Puerto Rican, based on a 24-h dietary recall suggested that the main source of sugar was sweetened beverages and 100 % fruit juices which were mainly consumed as afternoon snacks [22]. Another study also suggested that dental caries depends more on the frequency of carbohydrate intake than the quantity taken because frequency increases the duration for which it stays in the mouth, e.g., eating a bar of chocolate at one time is less damaging than eating it at intervals [21].

Duration of consumption of sugary drinks or foods means how long adults keep the sugary drinks or foods in their mouths before swallowing. Quick consumption is the habit of gulping food or drinks in a moment. Slow consumption is the habit of taking time while properly chewing the food or swishing drinks before swallowing. The current findings suggest no significant association between duration [slow or quick] of consumption of sugary foods or carbonated drinks and dental caries which means that dental caries was not associated with whether the sugary foods or drinks were taken quickly or slowly. This contrasts with a review by Gupta et al. who suggested that the critical factor in the advancement of caries depends on the duration for which the teeth are exposed to sugary foods rather than simply the form of the food [23].

The participants of the present study from both cases and controls had good oral hygiene. Findings showed no significant association between frequency and timings of brushing with dental caries. This suggests that adults with good oral hygiene from both groups brush their teeth with almost equal frequency and preferred timings. A secondary data output shows that many participants were still able to maintain adequate oral hygiene even with daily brushing once and at any time of the day. According to the findings, effective brushing is more important for maintaining good oral hygiene than how frequently and when it is done. A thorough review of the literature indicates that no studies have been published about the relationship between good oral hygiene and effective brushing as opposed to frequency and timing of brushing. Therefore, there has not been a comparable study available. This is a way forward for the future research.

In the current investigation, there was no discernible link between refined sugar and adults with or without caries. Although it is well known that children who consume more refined sugar have a higher incidence of dental caries [[24], [25], [26], [27], [28]], the current study found no evidence of this association among adults. The main objective of most researchers may be to determine how harmful sugar is to health, which could be the cause. Adults avoid consuming substantial amounts of refined sugar because they are more aware of the consequences it has on developing dental caries and other health problems, including diabetes and cardiac failure. Numerous investigations [29,30] that demonstrated no significant association between dietary consumption of sweets, candies, sugar, or snacks and the development of new caries concur with the current findings. Loveren in 2019, hypothesized that there was no linear or log-linear relationship between sugar consumption and dental caries. Additionally, he suggested that using fluoride toothpaste twice a day will greatly or almost eliminate the connection [31].

The current investigation found no relation between carbonated beverages and dental caries. Cohort research that examined children aged 4 with cavitated caries did not determine a significant association between dental caries and the use of carbonated beverages and soft drinks [32]. According to the present research, there is no connection between fruit juice consumption and tooth decay. On the other hand, Huew R. et al. Discovered a strong link between the consumption of sugary foods and beverages and dental caries [28]. The connection between sugar consumption and caries risk with fluoride intake was shown to be substantially weaker than it had previously been in a systematic review [29].

The Mann-Whitney test revealed a statistically significant difference in tea consumption between adults with and without dental caries. The adults of the present study preferred mixed tea i.e., tea with milk and added sugar. This is also corroborated by a study that suggested a high correlation existed between the severity of dental caries and the consumption of tea with sugar [28]. As opposed to this, a study on the relationship between tea and dental caries revealed that tea has an anti-cariogenic effect due to the presence of antioxidant Polyphenol and the tea's inhibitory effect on salivary amylase [32].

There are several limitations to this study, for example, the sample size for the present study was determined by the prevalence of dental caries in India, a neighboring country, because Indians and Pakistanis have similar lifestyles. Unfortunately, there were no case-control studies available among adults to compare the relationship between diet and dietary habits and dental caries, and the data on the prevalence of dental caries among adults in Pakistan was too old. Secondly, the study group was predominately educated and belonged to a middle-class socioeconomic level, therefore the conclusions were specific to that community rather than being generalized. Thirdly, dental caries is a phenomenon that takes time to establish. After experiencing dental caries, adults might have modified their diet and habits. So, the diet and dietary habits might differ from the actual diet when tooth decay started. Thus, the temporal relationship between the supposed cause and effect cannot be determined from this case-control study. Lastly, it was hospital-based convenience sample research due to financial constraints. However, the authors relentlessly found the association between diet and dietary habits among adults and dental caries, which provides fundamental data for future research.

The current investigation retained the null hypothesis that there is no association between adults’ dental caries and diets like refined sugar, fruit juices, and carbonated beverages. The current study also supported the null hypothesis that there is no association between dental caries and dietary habits like how often and when the brushing is done in a day as well as the duration of consumption of sugary food. However, it rejected the null hypothesis regarding the relationship between dental caries and dietary practices like snacking and smoking. Tea with added sugar, Frequent snacking, and smoking were found to be associated with dental caries. According to the current study, dental caries may affect more males than females. The current study's Cronbach's alpha value is 0.814, almost the same as that of the source study, which had a Cronbach's alpha value of 0.80 [11], indicating that the current study has very good reliability.

## 5 Conclusion

Dental caries is more closely linked to habits such as frequent consumption of sugar-enriched foods and added refined sugar in tea, consumed preferably as snacks between regular meals. Males were found to be more caries-affected than females. Additionally, smoking was found to be associated with dental caries.

## Research ethics approval

All human studies have been reviewed by the appropriate ethics committee of Rawal Institute of Health Sciences, Islamabad, Pakistan. It was performed following the ethical standards laid down in an appropriate version of the Declaration of Helsinki. This research received ethics approval under reference number RIHS/IRB/19–005.

## Funding

This research received no specific grant from any funding agency in the public, commercial, or not-for-profit sectors. This project was not funded by any institute or organization.

## Data availability statement

The data is not publicly available due to privacy and ethical restrictions. The data that support the findings of this study will be available upon reasonable request from the corresponding author.

## CRediT authorship contribution statement

**Kiran Javed:** Writing – original draft, Validation, Software, Resources, Project administration, Methodology, Investigation, Formal analysis, Data curation, Conceptualization. **Muhammad Zubair Nasir:** Writing – review & editing. **Maham Jalees:** Writing – original draft, Resources. **Manzoor Ahmed Manzoor:** Visualization, Supervision, Data curation.

## Declaration of competing interest

The authors declare that they have no known competing financial interests or personal relationships that could have appeared to influence the work reported in this paper.

## Footnotes

Appendix A Supplementary data to this article can be found online at https://doi.org/10.1016/j.heliyon.2023.e23117.
