# Supplementary material for: Role of diet and dietary habits in causing dental caries among adults reporting to a tertiary care hospital in Pakistan; a case-control study
Source: Heliyon. 2023 Nov 30;9(12):e23117. doi: 10.1016/j.heliyon.2023.e23117 (PMC10746458; doi:10.1016/j.heliyon.2023.e23117)
Supplement: Multimedia component 1 [file mmc1.pdf]

Rawal Institute of Health Sciences

RIHS/IRB 19-005

APPROVAL OF STUDY PROJECT BY ETHICS COMMITTEE

Rawal Institute of Health Sciences

Title:- **Role of diet and dietary habits in causing dental caries among adults reporting to tertiary hospital; A case-control study.**

Supervisor:
Prof. Dr. Brig (R) Manzoor Ahmad Manzoor
Head Department of Operative Dentistry
RIHS Islamabad

Candidate:
Dr. Kiran Javed
FCPS-II Resident
Operative Dentistry, RIHS

1. President Ethics Committee: Approved ☑ Not Approved ☐

Prof. Dr. Brig (R) Manzoor Ahmad Manzoor
Principal & Head Department of Operative Dentistry
Rawal Dental College, Islamabad

Brig (R)
Prof. Dr. Manzoor Ahmed
BDS, MCPS, FCPS, FICD
Supervisor / HOD
Operative Dentistry Department
Rawal Institute of Health Sciences
Islamabad

2. Member Ethics Committee: Approved ☑ Not Approved ☐

Dr. Farooq Kamran
(Head Department of Prosthodontics, RIHS)

3. *Member Ethics Committee:* Approved ☑ Not Approved ☐

Prof. Dr. Sadia Noureen
(Head Department of Orthodontics, RIHS)

4. Member Ethics Committee: Approved ☑ Not Approved ☐

Dr. Amna Muzafar
(Head Department of Oral Surgery, RIHS)

5. Member Ethics Committee: Approved ☐ Not Approved☐

Dr. Resham Hafeez
(Head of Department of Periodontology, RIHS)

6. Member Ethics Committee: Approved ☑ Not Approved ☐

Khateeb Ahmed Hussain (RIHS)

Brig (R)
Prof. Dr. Manzoor Ahmed
BDS, MCPS, FCPS, FICD
Supervisor / HOD
Operative Dentistry Department
Rawal Institute of Health Sciences
Islamabad

Scanned with CamScanner
