# Supplementary material for: Role of diet and dietary habits in causing dental caries among adults reporting to a tertiary care hospital in Pakistan; a case-control study
Source: Heliyon. 2023 Nov 30;9(12):e23117. doi: 10.1016/j.heliyon.2023.e23117 (PMC10746458; doi:10.1016/j.heliyon.2023.e23117)
Supplement: Multimedia component 2 [file mmc2.docx]

# APPENDIX 2

## **Participants Proforma**

Name ……………………………………………………………………………………….

Age …………………………………………………………………………………………..

Gender ……………………………………………………………………………………..

Contact Number ……………………………………………………………………….

Address …………………………………………………………………………………….

Socio Economic Status ………………………………………………………………

Estimated monthly income…………………………………………………………

Number of dependents in the family …………………………………………

Rented/ own house and car ………………………………………………………

Adult with Caries 🗌 Adult without Caries 🗌

Do you smoke 🗌

Do you take alcohol 🗌

Do you take tobacco, betel, betelnut 🗌

Do you take snuff 🗌

Factors that have demonstrated significant potential for causing caries:

- DURATION TO CONSUME SUGARY FOODS/ DRINKS:

Quickly/ Slowly

- WHEN THE FOOD WITH ADDED SUGAR IS TAKEN

During the meal _______ Yes/ No

Between the mealtimes _______ Yes / No

- BRUSHING PRACTICE

Miswak 🗌 How many times a day? ………. Timings

Toothbrush 🗌 How many times a day? ………. Timings

Finger 🗌 How many times a day? ………. Timings

Tooth Paste 🗌 How many times a day? ………. Timings

Mouth Wash 🗌 How many times a day? ………. Timings
