# Supplementary material for: Role of diet and dietary habits in causing dental caries among adults reporting to a tertiary care hospital in Pakistan; a case-control study
Source: Heliyon. 2023 Nov 30;9(12):e23117. doi: 10.1016/j.heliyon.2023.e23117 (PMC10746458; doi:10.1016/j.heliyon.2023.e23117)
Supplement: Multimedia component 3 [file mmc3.pdf]

APPENDIX 3

Food frequency questionnaire to compare the food frequency of adults with and without dental caries

FOOD		Quantity	Never or less than once/ month	1-3 per month	Once a week	2-4 per week	5-6 per week	Once a day	2-3 per day	4-5 per day	6 per day
Refined sugar	SUGAR										
	ICECREAM										
	CHOCOLATE										
	MITHAI/ HALWA										
	CANDIES/ JELLIES										
	JAM										
	BISCUITS										
	CAKE/ MUFFINS/ PASTRIES/ DOUGHNUTS										
	BREATH MINTS										
	CHEWING GUMS (with sugar)										
Drinks	FRUIT JUICES										
	FIZZY DRINKS										
	MIXED TEA										
	OTHER FOOD (not mentioned)										
